# Electrocardiographic predictors of peripartum cardiomyopathy

**DOI:** 10.5830/CVJA-2015-092

**Published:** 2016

**Authors:** Kamilu M Karaye, Kamilu M Karaye, Krister Lindmark, Michael Y Henein, Krister Lindmark, Michael Y Henein

**Affiliations:** Department of Medicine, Bayero University and Aminu Kano Teaching Hospital, Kano, Nigeria; Department of Public Health and Clinical Medicine, Umea University, Sweden; Department of Public Health and Clinical Medicine, Umea University, Sweden; Department of Public Health and Clinical Medicine, Umea University, Sweden; Department of Cardiology, Umea Heart Centre, Umea, Sweden; Department of Cardiology, Umea Heart Centre, Umea, Sweden

**Keywords:** peripartum cardiomyopathy, electrocardiogram, predictors, risk score

## Abstract

**Objective:**

To identify potential electrocardiographic predictors of peripartum cardiomyopathy (PPCM).

**Methods:**

This was a case–control study carried out in three hospitals in Kano, Nigeria. Logistic regression models and a risk score were developed to determine electrocardiographic predictors of PPCM.

**Results:**

A total of 54 PPCM and 77 controls were consecutively recruited after satisfying the inclusion criteria. After controlling for confounding variables, a rise in heart rate of one beat/minute increased the risk of PPCM by 6.4% (*p* = 0.001), while the presence of ST–T-wave changes increased the odds of PPCM 12.06-fold (*p* < 0.001). In the patients, QRS duration modestly correlated (*r* = 0.4; *p* < 0.003) with left ventricular dimensions and end-systolic volume index, and was responsible for 19.9% of the variability of the latter (*R*^2^ = 0.199; *p* = 0.003). A risk score of ≥ 2, developed by scoring 1 for each of the three ECG disturbances (tachycardia, ST–T-wave abnormalities and QRS duration), had a sensitivity of 85.2%, specificity of 64.9%, negative predictive value of 86.2% and area under the curve of 83.8% (*p* < 0.0001) for potentially predicting PPCM.

**Conclusion:**

In postpartum women, using the risk score could help to streamline the diagnosis of PPCM with significant accuracy, prior to confirmatory investigations

## Objective

Although peripartum cardiomyopathy (PPCM) was first described in 1880, much remains unknown about it.^1^,^2^ Electrocardiography (ECG) is an inexpensive and important tool for evaluating cardiac electrical function and is widely available, even in limited-resource settings. A recent study found the majority (96%) of PPCM patients had ‘abnormal’ 12-lead ECGs at presentation and highlighted the usefulness of the ECG in screening and prognosticating patients at risk in resource-poor settings.^3^ To the best of our knowledge, there is a paucity of ECG data in PPCM, and no data on its use in the diagnosis of PPCM in women presenting with clinical features of heart failure towards the end of pregnancy or during the puerperium.

The aim of this study was to determine potential ECG variables that predict the diagnosis of PPCM. If proved, such variables could help to streamline the diagnosis of PPCM prior to confirmatory investigations, particularly in limited-resource settings.

## Methods

This was a case–control study carried out in the Murtala Mohammed Specialist Hospital (MMSH), Aminu Kano Teaching Hospital (AKTH), and a private cardiology clinic in Kano, Nigeria. The research protocol was approved by the ethics committees of each of the study centres, and the study conformed to the ethics guidelines of the Declaration of Helsinki, on the principles for medical research involving human subjects.^4^

The inclusion criteria for the patients were: (1) confirmed diagnosis of PPCM; (2) onset of symptoms towards the end of pregnancy or within the puerperium, and presentation to hospital within nine months postpartum; (3) age of at least 18 years; and (4) written informed consent. Patients were excluded if: (1) their symptoms could be explained by diagnoses other than PPCM; (2) their symptoms started in early pregnancy or after the first five months postpartum; (3) they were younger than 18 or older than 45 years; (4) they denied consent to participate.

To be included, the controls had to satisfy the following criteria: (1) be apparently healthy; (2) no past history of any cardiac disease or systemic hypertension (except pregnancyinduced hypertension); (3) normal ECG (except for flat T waves in leads III or aVF, and inverted T waves in aVR, V1 or V2, which are considered non-specific);^5^ (4) present to the study centres within nine months postpartum for routine immunisations for their children; and (5) give written informed consent. Subjects taking drugs known to affect ECG intervals were excluded from the study.^6^

T-wave inversion with or without ST-segment depression were considered abnormal in all leads except aVR, V1 and V2.5 In addition, flat T waves in leads III or aVF were also considered non-specific.^5^

Controls were excluded if: (1) they presented their children for immunisation after five months postpartum; (2) they presented to the hospital as patients; (3) they were younger than 18 or older than 45 years; (4) they were known or found clinically to have any cardiac disease; (5) they denied consent. The sample size for the controls was estimated at 1.5 × the total number of patients (± five patients).

PPCM was defined according to the recommendations of the Heart Failure Association of the European Society of Cardiology working group on PPCM, and left ventricular (LV) systolic dysfunction was defined as LV ejection fraction < 50%.^7^

At the study centres, physicians in the Internal Medicine, and Obstetrics and Gynaecology Departments were approached and requested to refer all patients with suspected PPCM to the principal investigator (PI) for further evaluation. Patients were then interviewed, clinically evaluated and recruited consecutively. Hospital in-patients with PPCM were clinically assessed and underwent investigations within the first 48 hours of admission.

Demographic data, relevant aspects of the history and physical signs, results of investigations, co-morbid conditions, and complications were included in a detailed questionnaire. Baseline levels of serum urea, electrolytes and creatinine were carried out in the laboratories of AKTH, while 12-lead ECGs at rest, and transthoracic echocardiograms (for PPCM patients only) were all carried out by the PI at the study sites, according to standard recommendations.^8^ The echocardiographic examination was performed using a Sonoscape S8 Doppler ultrasound (Shenzhen, China, 2010) and the ECG was recorded using a Mindray DECG-03A digital electrocardiograph (Shenzhen, China, 2008).^8^,^9^

All ECG recordings were studied and interpreted by the investigators in the standard fashion, and ECG intervals/ durations were measured using manual callipers.^10^,^11^ The controls were evaluated using the same protocol as the patients, including the ECGs, but an echocardiogram was not performed.

## Statistical analysis

Frequencies, mean, median and inter-quartile ranges were used to describe patients’ characteristics. Chi-square, Fisher’s exact probability, Student’s *t*- and Mann–Whitney *U*-tests were used to compare categorical and continuous variables as appropriate. Binary logistic regression models were used to determine predictors of PPCM among the ECG variables, and values were expressed as odds ratios (OR) and 95% confidence intervals (CI). Pearson’s correlation coefficient and linear regression models were used to further assess relationships between variables of interest.

A simple score assigning 1 to each identified independent ECG predictor was composed and its accuracy in predicting PPCM was determined using the area under the receiver operating characteristics (ROC) curve (AUC), and AUC > 0.75 was considered satisfactory. A *p*-value < 0.05 was considered statistically significant. The statistical analysis was carried out using SPSS version 16.0 software.

## Results

A total of 54 PPCM and 77 controls satisfied all the inclusion criteria and were consecutively recruited. PPCM patients were recruited at the time of confirmation of diagnosis, when specific heart failure treatment was also commenced.

The baseline characteristics of the subjects are presented in Table 1. The mean age, body mass index (BMI), systolic (SBP) and diastolic blood pressure (DBP), and prevalence of pregnancy-induced hypertension were not significantly different between the two groups (*p* > 0.05). However, mean serum level of creatinine was higher (*p* = 0.045), and mean serum sodium and potassium levels were significantly lower (*p* = 0.009 and < 0.001 respectively) in patients compared to controls.

**Table 1:** Baseline characteristics of PPCM patients and controls

**	*PPCM patients (n = 54)*	*Controls (n = 77)*	*p-value*
Mean age (years)	26.6 ± 6.7	25.7 ± 5.7	0.450
Body mass index (kg/m^2^)	21.6 ± 4.3	21.8 ± 4.3	0.836
Systolic BP (mmHg)	119 ± 24	123 ± 16	0.293
Diastolic BP(mmHg)	86 ± 18	82 ± 12	0.099
Pregnancy-induced hypertension, n (%)	16 (41.0)	14 (28)	0.197
Serum creatinine (μmol/l)	93.2 ± 67.1	74.7 ± 19.3	0.045*
Serum sodium (mmol/l)	136.9 ± 5.9	139.6 ± 4.4	0.009*
Serum potassium (mmol/l)	3.9 ± 0.8	4.6 ± 0.7	< 0.001*

*p-value statistically significant; values are expressed as means ± standard deviations or as numbers with percentages in parentheses.

ECG findings are presented in Table 2. All subjects were in sinus rhythm, and ectopic beats and PR interval were not significantly different (*p* > 0.05) between the two groups. However, patients had significantly faster heart rates, broader QRS durations, prolonged QTc intervals, and more frequent tachycardia and ST–T-wave abnormalities (T-wave inversion with or without ST-segment depression in all leads except aVR, V1 and V2) than the controls (*p* < 0.004 for all comparisons).

**Table 2:** ECG features of PPCM patients and controls)

	*PPCM patients (n = 54)*	*Controls (n = 77)*	*p-value*
Sinus rhythm, n (%)	54 (100)	77 (100)	
Premature ventricular or atrial extrasystoles, n (%)	5 (9.3)	4 (5.2)	0.365
Heart rate, beats/min	111 ± 16	90 ± 16	< 0.001*
Tachycardia, n (%)	36 (66.7)	17 (22.1)	< 0.001*
QRS duration (ms)	109.9 ± 23.6	98.6 ± 12.8	0.004*
QRS duration ≥ 110 ms	19 (35.2)	8 (10.4)	0.001*
QTc duration (ms)	445.0 ± 34.2	421.2 ± 18.9	< 0.001*
QTc duration ≥ 460 ms	12 (22.2)	3 (3.9)	0.001*
PR interval (ms)	148.1 ± 20.4	149.1 ± 21.1	0.799
ST–T-wave abnormalities	37 (68.5)	13 (16.9)	< 0.001*

*p-value statistically significant; values are expressed as means ± standard deviations or as numbers with percentages in parentheses.

## ECG predictors of PPCM

The results of the logistic regression models are presented in Table 3. In the univariate analysis, heart rate, ST–T-wave abnormalities, and QRS and QTc durations were all predictors of PPCM (*p* ≤ 0.003). In addition, heart rate < 100 beats/min reduced the risk of having PPCM by 89.7% (*p* < 0.001). The presence of ST–T-wave abnormalities increased the odds of PPCM almost 12-fold (*p* < 0.001), while QRS duration > 110 ms and QTc duration > 460 ms increased the odds 5.2-fold (*p* < 0.001) and 9.5-fold (*p* < 0.001), respectively.

**Table 3:** Binary logistic regression models for predictors of PPCM

*Variables*	*Odds ratio*	*95% CI*	*p-value*
Univariate analysis			
ECG heart rate, beats/min	1.078	1.048–1.109	< 0.001*
QRS duration, ms	1.038	1.013–1.065	0.003*
QTc interval, ms	1.036	1.019–1.054	< 0.001*
Normal heart rate	0.103	0.044–0.241	< 0.001*
QRS ≥ 110 ms	5.241	2.057–13.355	0.001*
QTc ≥ 460 ms	9.471	2.548–35.199	0.001*
ST–T-wave abnormalities	11.970	5.160–22.770	< 0.001*
Multivariate analyses			
A (included variables: heart rate, QRS, QTc, ST–T-wave abnormalities)			
ECG heart rate, beats/min	1.073	1.036–1.112	< 0.001*
ST–T-wave abnormalities	14.591	4.581–46.480	< 0.001*
QRS duration, ms	1.028	0.994–1.062	0.105
QTc interval, ms	1.014	0.993–1.035	0.202
B (included variables: heart rate, ST-Twave abnormalities, serum potassium level)			
ECG heart rate, beats/min	1.066	1.029–1.104	< 0.001*
ST-T-wave abnormalities	12.056	3.507–4.443	< 0.001*
C (included variables: heart rate, ST-Twave abnormalities, serum sodium level)			
ECG heart rate, beats/min	13.415	4.203–42.825	< 0.001*
ST–T-wave abnormalities	1.064	1.029–1.101	< 0.001*

*p-value statistically significant; ECG, electrocardiogram.

Stepwise multivariate regression analyses were then carried out to control for confounding factors. In the initial model, including heart rate, ST–T-wave abnormalities, QRS duration and QTc interval, the former two maintained their statistical significance (*p* < 0.001 each), while the latter two lost their prediction of PPCM (*p* > 0.05 for each). The addition of serum sodium or potassium levels to the models with heart rate and ST–T-wave abnormalities did not influence the results, as the two ECG variables maintained their predictive value (*p* < 0.001), although serum potassium exerted greater influence than sodium levels. Therefore after controlling for confounding variables, including serum sodium and potassium levels, a rise in heart rate of one beat/min increased the risk of PPCM 6.4% (*p* = 0.001), while the presence of ST–T-wave changes increased the odds of PPCM 12.06-fold (*p* < 0.001).

## Relationship between ECG and echocardiographic variables

**Fig. 1.**
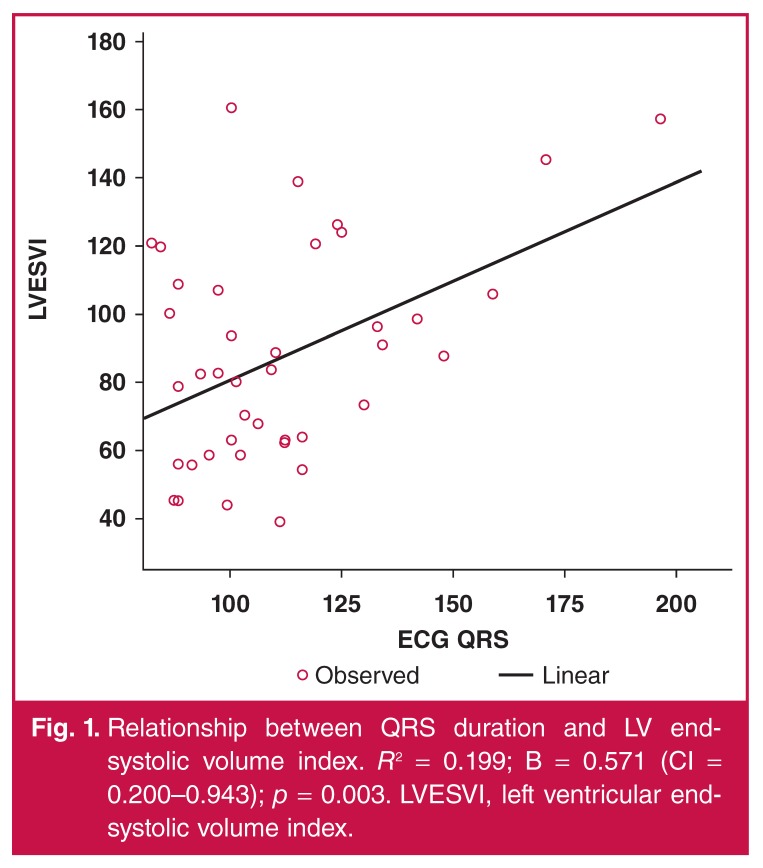
Relationship between QRS duration and LV endsystolic volume index. *R*^2^ = 0.199; B = 0.571 (CI = 0.200–0.943); *p* = 0.003. LVESVI, left ventricular endsystolic volume index.

The patients were evaluated echocardiographically. Mean end-diastolic and end-systolic dimensions (LVEDD and LVESD, respectively), LV end-systolic volume index (LVESVI) and LV ejection fraction (LVEF) were 61.4 ± 8.8 mm, 51.0 ± 9.1 mm, 85.1 ± 33.1 ml/m2 and 34.4 ± 9.9%, respectively. The relationship between these echocardiographic and ECG variables are presented in Table 4, which shows that QRS duration was the only variable that modestly correlated with LV dimensions and LVESVI, and showed a trend towards a significant relationship with LVEF (*r* = –0.27; *p* = 0.065). Fig. 1 shows the relationship between QRS duration and LVESVI, which was responsible for 19.9% of its variability (*R*^2^ = 0.199; *p* = 0.003).

**Table 4:** Correlation between ECG and echocardiographic variables

**	**	*ST–T-wave abnormalities*	*ECG QRS*	*ECG QT*	*ECG HR*
LVESD	Pearson correlation	+0.033	+0.446	+0.096	+0.109
	p-value	0.822	0.002*	0.515	0.460
LVEDD	Pearson correlation	+0.072	+0.420	+0.073	+0.039
	p-value	0.624	0.003*	0.624	0.793
LVEF	Pearson correlation	+0.015	–0.268	+0.009	–0.231
	p-value	0.920	0.065	0.949	0.115
LVESVI	Pearson correlation	+0.012	+0.446	+0.065	+0.095
	p-value	0.936	0.003*	0.685	0.553

*p-value statistically significant; ECG, electrocardiogram; HR, heart rate; LVESD, left ventricular end-systolic dimension; LVEDD, left ventricular end-diastolic dimension; LVEF, left ventricular ejection fraction; LVESVI, left ventricular end-systolic volume index.

## ECG risk score for PPCM

**Fig. 2.**
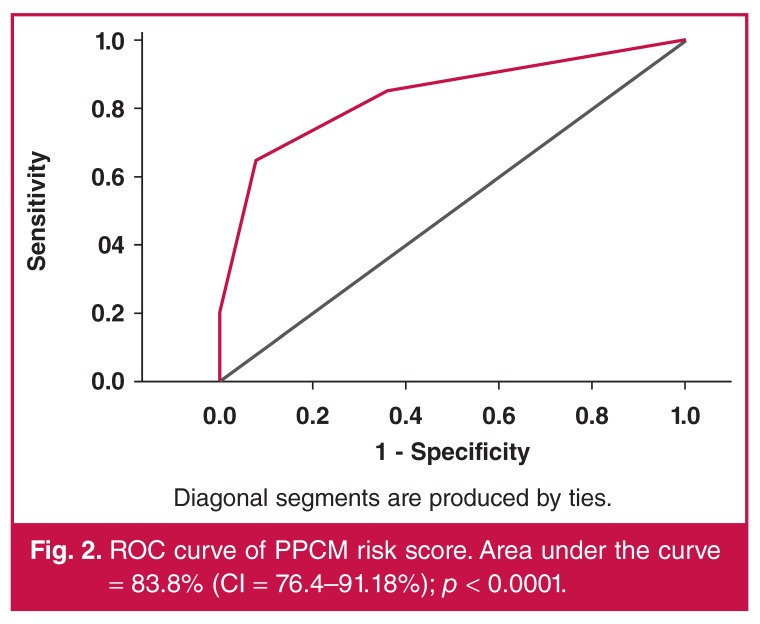
ROC curve of PPCM risk score. Area under the curve = 83.8% (CI = 76.4–91.18%); *p* < 0.0001.

Three variables, namely tachycardia, ST–T-wave abnormalities and QRS duration, were included in the risk score, counting 1 for each if present and 0 if absent (see Table 5). Tachycardia was defined as heart rate > 100 beats/min, ST–T-wave abnormalities as T-wave inversion with or without ST-segment depression in all leads except aVR, V1 and V2, and broad QRS duration > 110 ms. A total of 46 patients and 27 controls had a score of ≥ 2. This score had a sensitivity of 85.2%, specificity of 64.9%, positive predictive value (PPV) of 67.7%, negative predictive value (NPV) of 86.2% and AUC of 83.8% (CI = 76.4–91.2%; *p* < 0.0001) (see Fig. 2) for predicting PPCM.

**Table 5:** Demographic data of the study population (n = 118)

*ECG variable*	*Value*	*Score*
Heart rate, beats/min	< 100	0
	≥ 100	1
ST–T-wave abnormalities	Absent	0
	Present	1
QRS duration, ms	< 110	0
	≥ 110	1

## Discussion

This study describes, perhaps for the first time, the use of postpartum ECG variables in a simple risk score that seems to predict PPCM diagnosis among women at risk for the disease. PPCM patients and controls had similar age, systolic and diastolic blood pressures and body mass index. Therefore, the findings could not have been influenced by these possible confounders.

Our findings show that the presence of heart rate less than 100 beats/min reduced the risk of diagnosing PPCM to 89.7%, while the presence of ST–T-wave abnormalities, QRS duration more than 110 ms and QTc duration longer than 460 ms increased the odds of PPCM 12.0-, 5.2- and 9.5-fold, respectively. In the initial multiple regression model, heart rate and ST–T-wave abnormalities maintained their high statistical significance in predicting PPCM but not QRS duration and QTc interval. This finding was not influenced by serum sodium or potassium levels.

None of our patients had malignant arrhythmia, and ectopic beats were equally uncommon in both groups. These results are supported by previous reports in women with PPCM as well as in healthy pregnant women.^3^,^12^

Serum sodium and potassium levels were significantly lower in the patients than the controls, possibly because of water retention caused by heart failure syndrome.^7^ It should be noted that patients were all recruited at the time of confirmation of the diagnosis, when most heart failure treatments were commenced, and none was on drugs that could affect ECG measurements.

For the first time, we have developed a simple scoring system using three ECG variables that could potentially predict PPCM with an accuracy of 83.8%. A risk score of ≥ 2 had a sensitivity of 85.2%, specificity of 64.9% and NPV of 86.2%. The score’s satisfactory accuracy in predicting the diagnosis of PPCM makes it appealing for routine use, particularly in areas where the disease is prevalent and more expensive diagnostic facilities are limited.

Our results have shown that in the patients, ECG measurements were related to cardiac structure and function, suggesting diffuse pathological changes. QRS duration modestly correlated with LVEDD, LVESD and LVESVI, explaining 19.9% of the variability of the latter, but had only a trend towards a relationship with LVEF. Furthermore, QRS duration of > 110 ms significantly increased the odds of PPCM 5.2-fold, in spite of the faster heart rate in patients compared to controls.

These results are supported by a previous study, which showed an association between QRS duration and mortality among predominantly male (98%), elderly Caucasians with moderate to severe LV systolic dysfunction caused by a variety of diseases (LVEF < 40%).^13^ Therefore in patients presenting with clinical features of heart failure, QRS duration > 110 ms could be used to suggest significant LV dilatation and systolic dysfunction, as well as a poor prognosis.

T-wave abnormalities were identified in 68.5% of our patients. Similar findings were reported by Ntusi *et al.* in dilated cardiomyopathy patients, with a lower prevalence in idiopathic (68.8%) compared to those with familial disease (87.5%), but there was no association between such electric disturbance and survival.^14^ It seems therefore that T-wave changes, which reflect disturbed repolarisation, may have a less detrimental effect on a patient’s survival compared with QRS duration, which, in most, mirrors the impact of systolic dysfunction.

In our series, QRS duration was the only ECG variable that correlated with LV dimensions and end-systolic volume index, while heart rate and ST–T-wave abnormalities had higher sensitivity in predicting PPCM. Longitudinal follow-up studies would determine the prognostic impact of ECG variables in the setting of PPCM.

Heart rate and ST–T-wave changes seemed to independently predict the diagnosis of PPCM. In addition, a simple score of ≥ 2, counting 1 for each of three ECG abnormalities (tachycardia, ST–T-wave abnormalities and QRS duration > 110 ms) had 83.8% accuracy for predicting PPCM in women at risk. These findings could help to filter out patients requiring additional investigations in areas with limited resources.

This study has some limitations. Firstly, serum levels of calcium and magnesium were neither assessed nor controlled for, but deficiencies of these electrolytes have not been reported to be common in PPCM patients.^7^ Secondly, PPCM patients were not directly compared with patients with other conditions, such as dilated cardiomyopathy, but we relied on published evidence; therefore the findings cannot be considered specific to PPCM. The sample size was small but our results are consistent and seem to provide evidence for using easily measured ECG variables to predict PPCM in women at risk of the disease. Finally, echocardiography was not performed on the controls. They were screened using clinical evaluation and ECG only, which can be used to identify healthy women after delivery.3 Our findings should be considered as representative of the early stage of the disease, while long-term electrical abnormalities remain to be determined.

## Conclusion

This study shows, for the first time, that in women presenting within the first nine months after delivery with symptoms of heart failure, heart rate and ST–T-wave abnormalities were potential predictors of a diagnosis of PPCM. QRS duration modestly correlated with LV dimensions and LVESVI. A simple ECG-based score could potentially predict the diagnosis of PPCM, a finding that could help to streamline the diagnosis of PPCM prior to confirmatory investigations, particularly in limited-resource settings, where the disease is common.
